# Connexins Signatures of the Neurovascular Unit and Their Physio-Pathological Functions

**DOI:** 10.3390/ijms23179510

**Published:** 2022-08-23

**Authors:** Nunzio Vicario, Rosalba Parenti

**Affiliations:** Department of Biomedical and Biotechnological Sciences (BIOMETEC), Section of Physiology, University of Catania, 95123 Catania, Italy

**Keywords:** intercellular communication, astrocytes, microglia, neuroinflammation, neurodegeneration, connexin 43

## Abstract

Central nervous system (CNS) homeostasis is closely linked to the delicate balance of the microenvironment in which different cellular components of the neurovascular unit (NVU) coexist. Intercellular communication plays a pivotal role in exchanges of signaling molecules and mediators essential for survival functions, as well as in the removal of disturbing elements that can lead to related pathologies. The specific signatures of connexins (Cxs), proteins which form either gap junctions (GJs) or hemichannels (HCs), represent the biological substrate of the pathophysiological balance. Connexin 43 (Cx43) is undoubtedly one of the most important factors in glia–neuro–vascular crosstalk. Herein, Cxs signatures of every NVU component are highlighted and their critical influence on functional processes in healthy and pathological conditions of nervous microenvironment is reviewed.

## 1. Introduction

Intercellular communication is a fundamental process of cellular homeostasis in living organisms in which coexisting cell types communicate with each other and the surrounding microenvironment, optimizing and adapting their functions to their context. On the one hand, this delicate homeostatic balance is modified by many factors which may lead to dysfunction and disease, ranging from cancer to degenerative disorders, if they create an unbalance in the microenvironment’s composition [1,2,3,4,5,6,7,8,9,10]. On the other hand, the microenvironment’s composition is overseen by factors that restore coexistence and, therefore, suitable physiological conditions. The modulation of these mechanisms is able to establish a homeostatic balance in favor of cellular life or to drive cells to a pathological state and death [11,12,13,14]. The central nervous system (CNS) includes a heterogeneous population of cells integrated in a complex communication network which guarantees homeostasis and a permissive milieu for the activity of nerve cells. Communication within and between different compartments typically relies on direct cell–cell coupling via gap junctions (GJs) and indirect cell–extracellular compartment communication via hemichannels (HCs).

**Figure 1:**
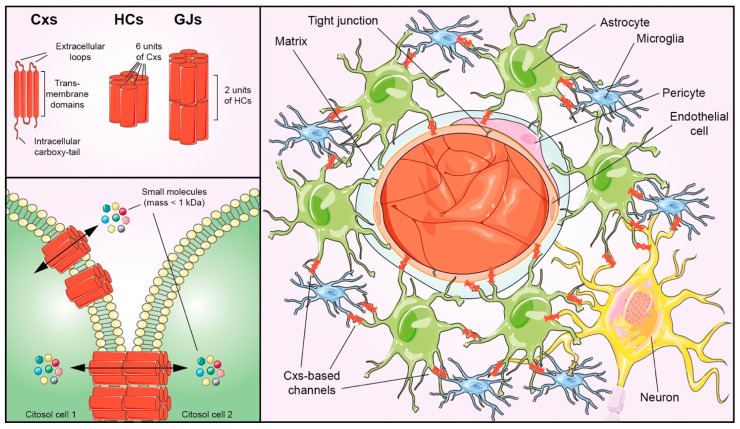
Schematic representation of Cxs-based HCs and GJs and NVU components. At the NVU level, blood vessels, composed of endothelial cells, are surrounded by a matrix and pericytes. Astrocytes, microglia, and neurons cooperate in the NVU to form complex hetero-cellular coupling, facilitating homeostasis and balance in the CNS. Cxs: connexins; HCs: hemichannels; and GJs: gap junctions.

Connexins (Cxs) are composed of four transmembrane domains, two extracellular loops, one intracellular loop and one intracellular carboxy-tail (Figure 1). They represent the core proteins of GJs and HCs. Each HC has six Cxs units; two HC units on the membrane of two adjacent cells form a GJ. Cxs’ composition qualifies homomeric and heteromeric HCs and homotypic and heterotypic GJs [15,16]. HCs allow exchanges between the intracellular compartment and the extracellular milieu.

There are at least 21 genes encoding Cxs in humans, each one named according to its theoretical molecular mass in kDa and with a specific expression profile in CNS cells [16,17,18]. In this regard, astrocytes mainly express Cx43, neurons express Cx36, oligodendrocytes and microglia express high levels of Cx32, and endothelial cells express Cx40 and Cx43; however, cells dynamically modify their Cx signature in response to specific stimuli or during pathological processes [16].

HCs and GJs allow molecules up to one kDa, including metabolites, nutrients, ATP, and second messengers, to pass through membranes. In addition to forming opened or closed pores, both structures represent selective frontiers based on their Cxs composition and are strictly modulated by a number of mediators, including voltage, pH, calcium level, kinase activity, metabolites, and signal molecules [19,20,21,22].

In this scenario, CNS cells, including neurons, glial cells, endothelial cells, and pericytes, form the so-called neurovascular unit (NVU), in which each component participates to orchestrate and maintain homeostasis in terms of ions composition, trophic factors, energy substrates, and removal of waste products [23,24,25]. The NVU is the core structure of the blood–brain barrier (BBB); physiologically, it acts as a highly selective barrier function, separating the CNS from the periphery, protecting the multicellular nerve framework from ionic fluctuations, and ensuring the elimination of brain metabolic waste [26,27,28]. The NVU is characterized by an efficient structural system based on a mutual and synergistic collaboration between its cellular components. Compelling evidence suggests that modulation of glial and neuronal GJs and HCs at the NVU level represents an efficient strategy to control permeability, blood flow, and metabolic trafficking, highlighting its potential therapeutic role for neurodegenerative diseases [26,27,28,29]. Thus, Cxs are primary contributors to the NVU’s critical role in the BBB’s operation.

This review highlights the *Cxs signatures* of NVU components (Figure 1) regarding their role in maintaining the CNS’s delicate homeostatic balance and in disease pathogenesis.

## 2. Connexins Signatures of NVU Components

Among the GJs’ and HCs’ functions in the CNS, the regulation of ions, mediators, and metabolites in the NVU is one of the most complex and inspiring [25,30,31]. NVU components interact during homo-cellular and hetero-cellular communication, involving a significant number of mediators and influencing related cell signaling processes. To share this complex function, specific Cxs profiles characterize NVU cell populations, including neurons, microglia, astrocytes, oligodendrocytes, brain microvascular endothelial cells (BMECs), smooth muscle cells (SMCs), pericytes, and the brain-specific extracellular matrix (ECM). Below, we review each of these components in relation to its Cxs signature, which influences the crosstalk underlying NVU homeostasis.

### 2.1. Neurons

Neuronal survival and physiological functions strictly depend on maintenance of the BBB structure. Indeed, disruption of physiological BBB permeability induces CNS suffering, tissue damage, and cell loss [32]. As neurons use most of the energy and substrates delivered to the CNS, intercellular communication with and between NVU cell populations, including astrocytes, microglia, and mural cells, is a critical regulator of energetic needs. This process is finely regulated by a mechanism that increases blood flow in response to decreased energy substrates [32] and a feed-forward regulation that increases metabolic waste removal, heat dissipation, and the supply of substrates in response to increased synaptic activity [33,34,35,36].

In particular, active synapses are strictly dependent on the network of astrocytes adapted to neuronal demand. In fact, a mutual control operates between neurons and the Cxs-based astrocyte network, with bidirectional influences on neuronal activity and the astroglial Cxs signature [37,38,39].

In recent decades, the scientific literature has extensively focused on neuron-specific Cxs and related GJs and HCs involved in fundamental functions in the CNS. During CNS development, compartmentalization of unitary cell groups following common developmental paths is critically determined by transient modulation of specific GJs activity [40,41]. In the adult brain, GJs control the electrical synchronization and response required for brain activity in specific areas via a close relationship with chemical transmission; GJs also function in response to different types of neuronal injuries [24]. It has been demonstrated that Cxs, together with pannexin-based HCs, induce electrical activity in precocious subplate neurons located between the intermediate zone and the cortical plate in the human fetal CNS; whereas in the adult CNS, Cx36 HCs are involved in ATP release, followed by activation of P2Y purinergic receptors and signal transduction pathways [42,43].

Cx36 is the main neuronal GJs- and HCs-forming protein; it is spatially and temporally regulated and involved in multiple functions [14,44,45,46]. However, the focus of this review is not the complexity of the neuronal network’s role in brain function, but rather the multifaceted crosstalk between glial components at the NVU level which functions as a dynamic sentinel in the cerebral microenvironment, becoming a master regulator of CNS functions.

### 2.2. Nerve Glial Cells

#### 2.2.1. Astrocytes

Astrocytes are considered critical players in maintaining tissue homeostasis and function within the CNS. Establishing an elaborate network, they have a primary function at the BBB level and finely regulate NVU activity [47,48,49]. Indeed, astrocytes regulate BBB permeability and overall functions through end foot-enveloping vascular endothelial cells and pericytes [50]. At the same time, through perisynaptic processes, astrocytes contribute to the formation of the so-called “tripartite synapse”, where they support and even modulate neuronal signaling activities, including neurotransmitters release, and the concentration, half-life, and transport of inositol 1,4,5-trisphosphate (IP3) and other gliotransmitters [51,52,53,54,55]. As such, astrocytes are central elements required to couple neuronal signaling and metabolic activities with cerebral blood flow. In particular, the functional and coordinated interactions of astrocytes with neurons and vascular cells guarantee appropriate vasomotor responses to the different metabolic needs of the microenvironment in which neurons act.

Astrocytic end feet are connected by GJs composed of Cx43 and Cx30 both temporally and spatially expressed [39,56,57]. The consequential homo-cellular (i.e., astrocyte–astrocyte) or hetero-cellular communication represents one of the crucial interaction checkpoints available to modulate NVU functions and CNS homeostasis [58]. Overall, to maintain the integrity of this complex network, astrocytes establish a close collaboration between integrins, Cxs, and different elements of the extracellular matrix [59]. In particular, neuronal activity, through the release of neurotransmitters, triggers a calcium signaling response in astrocytes that propagates to the astrocytic end feet, resulting in ATP release both through Cx30/Cx43- and pannexin1-based HCs [60]. This mechanism is finely regulated by nitric oxide, which may contribute to vasodilation of arterioles parenchyma [60]. Mutant mice lacking Cx30 and Cx43 have exhibited an altered neurochemical microenvironment, electrophysiological dysregulation, dysmyelinating phenotype, and general BBB weakness, which finally results in behavioral abnormalities [61,62,63,64].

The roles of Cx30 and Cx43 are likewise evident in the tripartite synapse, where they influence synaptic activity by regulating presynaptic glutamate levels and its transport [56,65,66,67]. Cx43 is one of the most studied and abundant Cxs; it is widely expressed by glial cells [68,69]. Cx43 expression in astrocytes has been associated with autocrine/paracrine signaling due to the release of nucleotides, ATP, and other mediators via HCs [70,71]. This establishes HCs as key players in the microenvironmental and paracrine modulation of NVU physiological and pathological control. Astrocytes also express high levels of Cx30, exert significant influence at the NVU level, and control BBB integrity and functions [63,72,73]. Nearby cells may modulate astrocytes’ function by either inducing or repressing Cx43 and Cx30 expression levels [37,74,75].

#### 2.2.2. Microglia

The contribution of microglia to the NVU is of particular importance in physiological processes ranging from CNS development to influencing cerebral blood flow and neuronal circuits including synaptic plasticity, learning, and memory [76,77]. This role determines the evolution of the ‘‘tripartite synapse’’ into a “quad-partite synapse’’ [78,79,80]. Indeed, microglia cells sense and influence homeostasis at the NVU level via anti-inflammatory and neurotrophic factors. They are also able to dynamically change phenotype to help restore homeostasis or become sources of detrimental effects in the case of a brain insult, depending on the insult’s intensity and chronicity [78,81,82].

Microglia’s Cxs signatures depend on the context in which they act [79]. In physiological conditions of surveillance, unpolarized microglial cells express Cx36 and Cx32 [79,83], whereas polarized microglial cells expand their Cxs signatures, actively expressing Cx29 [84], Cx43 [79,85,86], Cx32 [84,87], and Cx36 [83,88] in response to damaging stimuli. Thus, inflammatory microglia, via Cxs-based and pannexin-based channels, allow the activation of a cell signaling pathway, inducing stimulation of glutaminase and glutamate induced excitotoxicity, interleukin-1β (IL-1β) release, and increased extracellular ATP levels [79].

The balance between pro- and anti-inflammatory factors sustains physiological interactions between microglia and astrocytes, resulting in homeostatic restoration at the NVU level [89,90].

#### 2.2.3. Oligodendrocytes

Oligodendrocytes, which form myelin sheaths around axons, primarily contribute to the development of complex neural circuits by providing energy substrates to neurons, sustaining saltatory synaptic transmission and plasticity, and coordinating intercellular communication with neurons and other glial cells [91].

Oligodendrocytes’ Cxs signatures are dynamic and strictly regulated by crosstalk with glial cells [92]. Oligodendrocytes mainly express Cx32, Cx29, and Cx47 [92,93,94,95,96,97,98], participating in the formation of GJs between oligodendrocytes, between oligodendrocytes and astrocytes, or even between myelin layers of the same oligodendrocyte [99,100]. The complex ‘panglial’ network of oligodendrocytes and astrocytes is capable of spatially buffering potassium, water transport, and bi-directional calcium waves during neuronal activity [101,102]. Moreover, Cx–panglial substrates regulate metabolic demand through oligodendrocyte–astrocyte GJs and HCs activity, enabling glucose uptake and supporting oligodendrocyte precursor cells proliferation, which is triggered by spontaneous intracellular calcium signaling in both physiological and energy-related diseases [103].

In the NVU microenvironment, a close interdependence between oligodendrocytes and brain microvascular endothelial cells (BMECs) has been described. Endothelial cells can influence the survival and proliferation of oligodendrocyte precursor cells through the release of factors including brain-derived neurotropic factor (BDNF) [104,105]; in contrast, oligodendrocyte progenitors can support BBB integrity via transforming growth factor-β (TGF-β) signaling [106]. It has been reported that oligodendrocytes aid the NVU in protecting white matter and mediate remyelination of damaged white matter via NVU-based oligodendrocyte progenitor cells’ stimulation [105]. Evidence supports the hypothesis of a detrimental role of Cxs, particularly astroglial Cx43-based HCs, in preventing or inhibiting oligodendrocyte progenitor cells’ maturation [107].

### 2.3. Brain Microvascular Endothelial Cells (BMECs)

BMECs represent the first barrier from peripheral circulation; they regulate transport into and out of the CNS and to ensure protection and homeostasis. Their barrier function is guaranteed by a complex network of protein forming cell–cell junctions, including claudins, occludins, adherens junctions, tight junctions, and zonula occludens proteins. In particular, the literature extensively suggests that BBB integrity is highly dependent on Cxs-forming GJs and HCs between BMECs and other NVU components [108].

Through GJs, endothelial cells propagate vasoactive signals among themselves and/or with adjacent vascular cells, responding in a unitary manner to mechanical or chemical stimuli from astrocytes or neurons, and allowing adequate vascular response to the CNS’s metabolic needs [109,110,111,112].

Research regarding endothelial cells’ Cxs expression profile suggests that they constitutively express Cx37, Cx40, and Cx43; even if in homeostatic conditions, they express low Cxs levels in the NVU and increasing cell–cell or cell–extracellular communication in response to injury [113,114,115]. It is noteworthy that Cx40, Cx43, and particularly Cx37 are implicated in calcium homeostasis, regulating communication, and apoptosis [116,117].

The high capability of endothelial cells to dynamically modulate their Cxs expression profile and the permeability of the channels they are forming is significant. Indeed, even if no direct contact between astrocytes and endothelial cells occurs in the NVU, these cells can interact in an in vitro coculture system [57,118,119,120]. The capability of endothelial and glial cells to effect such plastic changes, shaped the idea that they are able to modulate intercellular communication, ion homeostasis, and paracrine mediators release in a context dependent manner [113,114,115].

Endothelial cells also control vascular endothelial growth factor (VEGF) levels throughout their Cxs-based channels [121]. Indeed, endothelial Cx43 levels positively correlate with VEGF; in pathological conditions, increased Cx43 levels in endothelial cells result in a reactive activation of nearby astrocytes, increased cell death and oxidative stress [122]. Interestingly, experimental evidence shows that Cx43 downregulation reduces stem cell differentiation and, vice versa, that VEGF increases Cx43 expression levels, accelerating endothelial repair [123,124]. This effect is coupled with increased GJs-based intercellular communication in astrocytes induced by endothelial-derived VEGF [121]. This scenario creates an intercellular loop in which Cx43 plays a central role; in fact, Cx43 reduction in endothelial cells reduces Cx43 levels in astrocytes and also reduces VEGF release and angiogenesis [125]. Moreover, a reduction in Cx43-based channels impairs endothelial cells-mediated regeneration. It is noteworthy that Cx43 expression levels are also modulated by Wnt and sonic hedgehog signaling pathways, both of crucial importance in developing and adult brains [126].

### 2.4. Pericytes

Pericytes, in close contact with BMECs, are the mural cell constituents of the NVU, representing the physical interface separating the CNS resident cells and the periphery. Pericytes play a significant role in BBB regulation, permeability, and angiogenesis, and act as a stem cells source, supporting regenerative processes in the adult brain [127,128,129]. Pericytes participate in the regulation of cerebral blood flow, making intermittent high coverage contact with capillaries and post-capillary venules [130,131].

Intercellular communication has been investigated with a particular focus on cell–cell coupling between pericytes and endothelial precursor cells, which is a critically importance process during neovascular formation. This mechanism is associated with Cx43-based GJs, with a significant loss of neo-angiogenesis in a Cx43-depleted brain [132]. Notably, this phenomenon has not been reported in all subjects lacking Cx43-based GJs in pericytes [132]. As such, it is likely that communication typically mediated by GJs still occurs via other Cxs, which compensates for the Cx43 loss.

Cx43-based channels, together with N-cadherin, support the so-called adhered phenotype of pericytes and endothelial cells [133]. PGE2 is known to induce a migrating phenotype in pericytes, mediating a breakdown of the pericyte–endothelial cell interaction and vascular destabilization [133]. Even though it has been demonstrated that pericytes actively express Cx43, the roles of other Cxs (such as Cx30), independent of their channel-forming properties, have been proposed and deserve further investigation [134].

Finally, pericytes’ functions are not restricted to NVU homeostasis; outside the NVU, they also regulate stem cells’ differentiation and fate [135,136].

### 2.5. Smooth Muscle Cells (SMCs)

Smooth muscle cells (SMCs) have contractile capability and are mural cells of the NVU, enveloping the endothelial layer of arterioles and venules. Together with pericytes and endothelial cells, SMCs regulate blood flow and BBB integrity, regulating the cerebrovascular tone of arteries and arterioles. Astrocytes and neurons directly communicate with SMCs, releasing vasoactive mediators in response to stimuli, such as glucose levels [137,138]. Further studies are needed to clarify whether such a regulatory effect induces either vasoconstriction or vasodilation [139,140,141].

Intercellular communication between SMCs and NVU cells relies on Cxs-based and pannexin-based channels which, acting as ATP and calcium channels, modulates vascular remodeling and cell migration [142]. Recent reports regarding SMCs Cxs signatures show that SMCs actively express Cx43-based channels, even if their role seems strictly context dependent and not fully elucidated (particularly their role in inhibiting SMCs autophagy) [143]. Suppression of Cx43 levels in SMCs increases cell proliferation and reduces cell death, supporting the hypothesis of a potential therapeutic strategy based on Cx43 targeting for inflammatory and vascular diseases affecting SMCs [144].

### 2.6. Brain-Specific Extracellular Matrix (BSECM)

The BSECM represents the noncellular anatomical substrate providing mechanical, structural, and biochemical support to the nervous components of the NVU [66,145]. It includes cell adhesion receptors, several proteins, glycosaminoglycan, and glycoconjugate interacting within a complex molecular network. A number of cell adhesions and matricellular molecules contribute to synaptic functioning through direct influence on all components of the tripartite synapse [146,147]. Of note, many have become therapeutic targets for treatment of CNS pathologies [146,147]. Most proteins, including laminin, proteoglycans, collagen isoforms, cadherins, and catenin, anchor cells to each other, functioning as attachment points for pericytes and endothelial cells [148,149,150,151]. In this context, through specific Cxs signatures, NVU components dynamically communicate with each other, enabling behavior, metabolic activity, and BBB integrity [146,148,152,153].

## 3. Overview and Concluding Remarks

The NVU is a representative example of the complex organization required to maintain a permissive microenvironment that allows physiological functions to take place. Intercellular communication plays a central role in all NVU functions by exchanging signaling molecules, adapting to disturbing elements, and sharing functional molecules. GJs and HCs are among the most important physiological substrates in this system, capable of turning the conditions of homeostatic maintenance toward increased resistance to injury, or compromised resistance to metabolic perturbation and oxidative stress. Their responses depend on the features of pathophysiological stimuli or different stages of the pathological process. For this critical dichotomy, in the past decades GJs have earned the definitions “kiss of death” and “kiss of life” [154,155,156].

In light of this, specific Cxs signatures of NVU components emerged as an attractive target to improve or counteract the chronicization or progression of associated diseases. If in some cases a specific Cxs’ dysregulation can be solved thanks to their functional mutuality, in other cases, unbalance and functional loss become inevitable and unbridgeable, leading to neurological disorders [157,158,159].

Neuroinflammation is a common feature of BBB related disease. Indeed, neuroinflammatory conditions, as a primary cause or as a consequence of the most common cerebral insults, are associated with different grades of involvement of Cxs-based GJs and HCs. The latter display opposite roles, being involved in maintaining BBB integrity (GJs) and in releasing ATP, modulating calcium, and sustaining inflammatory signals leading to BBB disruption (HCs). Accordingly, conditions including increased capillary permeability, metabolic changes, and glucose trafficking are closely linked to cellular exchanges via Cx43-HCs and a concomitant reduction in Cx43-GJs-mediated intercellular communication [19,23,160,161]. As synergic contributors to this condition, neurons and microglia trigger an activation/deactivation loop, regulating Cx43/Cx30 expression in astrocytes, and modulating endothelial cell functions [37,74,75]. This condition impacts permeability in paracellular and transcellular routes, thereby leading to vascular leakage, release of vasoactive substances, sustained neuroinflammation, and severe brain insults [114,115,162]. Reactive microglia support the release of IL−1β, interleukin−6 (IL−6), and tumor necrosis factor (TNF), which support an aberrant opening of astrocytic Cx43-HCs. Activated astrocytes sustain the release of inflammatory factors, including chemokines and cytokines; this propagates an inflammatory response, promoting the recruitment of leukocytes [19,160,163]. Moreover, neurons, prompted by the Cx43-based HCs-mediated release of gliotransmitters (ATP/glutamate), exhibit up-regulation of Cx36-HCs and a consequent calcium neuronal overload, resulting in structural neuronal alterations and increased oxidative stress. Finally, the persistence of the brain insult associated with the unbalance of Cx43-GJs and -HCs ultimately translates to reduced neuroprotection [19].

A number of neurodegenerative disorders and brain insults are linked to dynamic changes in HCs and GJs activity, which characterize neuroinflammatory conditions. In particular, Cxs/pannexins HCs expressed by astrocytes are reported to be involved in the release of soluble factors including glutamate, ATP, anaphylatoxins, TNF, apolipoprotein E (ApoE), and specific miRNAs. In turn, these molecular players are critical in the pathogenesis of neurodegenerative disorders, including multiple sclerosis [164,165], Alzheimer’s Disease [166], Amyotrophic Lateral Sclerosis [3,167,168,169], and others [170]. For instance, astroglial Cxs remodeling contributing to neuronal alterations has been observed in two different β-amyloid precursor protein (APP)/presenilin1 (PS1) murine models of Alzheimer’s Disease [166]. Both in vivo and in vitro models of ischemia/reperfusion injury have shown that suppression of abnormal astroglial Cx43 HCs openings by the Cx43 mimetic peptide Gap19 reduces Toll-like receptor 4 (TLR4) pathways as well as the accumulation and release of inflammatory cytokines, including TNF and IL-1β [171]. It has also been demonstrated that Gap19 induces JAK2 and STAT3 pathways in astrocytes; these effects can be reverted using selective JAK2/STAT3 pathway blockers [172].

Furthermore, in an experimental model of cerebral ischemia, it was shown that the protective effects of erythropoietin (EPO), which is capable of reducing BBB disruption and neuronal death, depend on a mechanism involving Cx43 phosphorylation, although the level of Cx43 was not modulated. In this case, the induction of GJs intercellular communication improved the clearance of neurotoxic mediators and excitotoxic stimuli (i.e., calcium and glutamate levels) [173]. Additionally, in a mouse ischemia/reperfusion model, it was demonstrated that administration of danegaptide (ZP1609), an antiarrhythmic dipeptide that specifically enhances GJs conductance, significantly reduces infarct volume by increasing Cx43–GJs coupling in astrocytes [174]. In an animal model of traumatic brain injury (TBI), it was observed that an impairment of platelet-derived growth factor-B (PDGF-B) signaling, resulting in a loss of pericyte–endothelium interaction and consequent neurovascular dysfunction, correlates with reduced levels of N-cadherin, adherent junction, and Cx43 functionality [175]. A noteworthy finding is the decline and/or reduction in Cx43–GJs along the arteriole–capillary vascular pathway; this is considered one of the first indicators of experimental diabetic retinopathy associated with reduced propagative vasomotor activity and cell coupling as well as compromised anatomical and physiological integrity of the retina [176].

Even if GJs and HCs are indicated as leading causes of chronicization and secondary damage during neuroinflammatory disease, it is not certain whether full ablation and/or inhibition of Cxs-based channels is appropriate in other conditions. Cx43 ablation in astrocytes after focal brain ischemia increases apoptosis and inflammation, suggesting that Cx43 ablation in astrocytes may result in a loss of their critical modulatory and neuroprotective function [177]. However, full ablation of Cx43-based channels significantly impacts intercellular communication in the CNS and at the injury site; solid data indicate a beneficial effect of homo-cellular homomeric Cx43-channels inhibition in neurovascular disease [178]. Moreover, ablation of Cx43 reduces neural progenitor cells’ ability to migrate, modulate the immune system, and eventually repair CNS damage [179,180,181,182]. More generally, neuroinflammation related to dysregulation of Cxs-forming GJs and HCs represents a cause/effect for multiple pathological conditions. Among these, the role of cell–cell and cell–extracellular environment communication described during nociceptive signaling in chronic neuropathies is noteworthy. This phenomenon leads researchers to study Cxs as a target for developing new analgesics strategies [15,183,184,185].

Finally, alterations and dysregulation of Cxs have been identified in brain tumors [159,186,187]. In particular, Cx43 plays a leading role in glioma in the process of invasion, progression, and resistance to temozolomide and radiotherapy [186,187,188,189,190].

In conclusion, the NVU’s delicate homeostatic balance is influenced by all NVU cellular components that exhibit specific Cxs signatures. It is equally true that a leading role must be assigned to Cx43-based channels that, acting as coordinators of this complex network, are dynamically modulated in terms of HCs- or GJs-forming proteins. Cx43′s functional versatility pushes the scientific community to pay it particular attention when planning new therapeutic strategies to improve clinical outcomes of NVU-related CNS disorders.

## Abbreviations

ApoE	Apolipoprotein E
APP	β-amyloid precursor protein
BBB	Blood brain barrier
BDNF	Brain-derived neurotropic factor
BMEC	Brain microvascular endothelial cell
BSECM	Brain-specific extracellular matrix
CNS	Central nervous system
Cx	Connexin
ECM	Extracellular matrix
EPO	Erythropoietin
GJ	Gap Junction
HC	Hemichannel
IL-1β	Interleukin-1β
IL-6	Interleukin-6
IP3	Inositol 1,4,5-trisphosphate
NVU	Neurovascular unit
PDGF-B	Platelet-derived growth factor-B
PS1	Presenilin1
SMC	Smooth muscle cell
TBI	Traumatic brain injury
TGF-β	Transforming growth factor-β
TLR4	Toll-like receptor 4
TNF	Tumor necrosis factor
VEGF	Vascular endothelial growth factor

## References

[1] Vicario N., Bernstock J.D., Spitale F.M., Giallongo C., Giunta M.A.S., Li Volti G., Gulisano M., Leanza G., Tibullo D., Parenti R. (2019). Clobetasol Modulates Adult Neural Stem Cell Growth via Canonical Hedgehog Pathway Activation. Int. J. Mol. Sci..

[2] Vicario N., Spitale F.M., Tibullo D., Giallongo C., Amorini A.M., Scandura G., Spoto G., Saab M.W., D’Aprile S., Alberghina C. (2021). Clobetasol promotes neuromuscular plasticity in mice after motoneuronal loss via sonic hedgehog signaling, immunomodulation and metabolic rebalancing. Cell Death Dis..

[3] Vicario N., Calabrese G., Zappala A., Parenti C., Forte S., Graziano A.C.E., Vanella L., Pellitteri R., Cardile V., Parenti R. (2017). Inhibition of Cx43 mediates protective effects on hypoxic/reoxygenated human neuroblastoma cells. J. Cell. Mol. Med..

[4] Camiolo G., Barbato A., Giallongo C., Vicario N., Romano A., Parrinello N.L., Parenti R., Sandoval J.C., Garcia-Moreno D., Lazzarino G. (2020). Iron regulates myeloma cell/macrophage interaction and drives resistance to bortezomib. Redox Biol..

[5] Tibullo D., Longo A., Vicario N., Romano A., Barbato A., Di Rosa M., Barbagallo I., Anfuso C.D., Lupo G., Gulino R. (2020). Ixazomib Improves Bone Remodeling and Counteracts sonic Hedgehog signaling Inhibition Mediated by Myeloma Cells. Cancers.

[6] Sanfilippo C., Castrogiovanni P., Imbesi R., Tibullo D., Li Volti G., Barbagallo I., Vicario N., Musumeci G., Di Rosa M. (2019). Middle-aged healthy women and Alzheimer’s disease patients present an overlapping of brain cell transcriptional profile. Neuroscience.

[7] Tibullo D., Giallongo C., Romano A., Vicario N., Barbato A., Puglisi F., Parenti R., Amorini A.M., Wissam Saab M., Tavazzi B. (2020). Mitochondrial Functions, Energy Metabolism and Protein Glycosylation are Interconnected Processes Mediating Resistance to Bortezomib in Multiple Myeloma Cells. Biomolecules.

[8] Bernstock J.D., Vicario N., Li R., Nan L., Totsch S.K., Schlappi C., Gessler F., Han X., Parenti R., Beierle E.A. (2020). Safety and efficacy of oncolytic HSV-1 G207 inoculated into the cerebellum of mice. Cancer Gene Ther..

[9] Torrisi F., Vicario N., Spitale F.M., Cammarata F.P., Minafra L., Salvatorelli L., Russo G., Cuttone G., Valable S., Gulino R. (2020). The Role of Hypoxia and SRC Tyrosine Kinase in Glioblastoma Invasiveness and Radioresistance. Cancers.

[10] Parenti R., Puzzo L., Vecchio G.M., Gravina L., Salvatorelli L., Musumeci G., Vasquez E., Magro G. (2014). Immunolocalization of Wilms’ Tumor protein (WT1) in developing human peripheral sympathetic and gastroenteric nervous system. Acta Histochem..

[11] Calabrese V., Dattilo S., Petralia A., Parenti R., Pennisi M., Koverech G., Calabrese V., Graziano A., Monte I., Maiolino L. (2015). Analytical approaches to the diagnosis and treatment of aging and aging-related disease: Redox status and proteomics. Free Radic. Res..

[12] Parenti R., Cicirata F., Panto M.R., Serapide M.F. (1996). The projections of the lateral reticular nucleus to the deep cerebellar nuclei. An experimental analysis in the rat. Eur. J. Neurosci..

[13] Panto M.R., Cicirata F., Angaut P., Parenti R., Serapide F. (1995). The projection from the primary motor and somatic sensory cortex to the basilar pontine nuclei. A detailed electrophysiological and anatomical study in the rat. J. Hirnforsch..

[14] Cicirata F., Parenti R., Spinella F., Giglio S., Tuorto F., Zuffardi O., Gulisano M. (2000). Genomic organization and chromosomal localization of the mouse Connexin36 (mCx36) gene. Gene.

[15] Vicario N., Turnaturi R., Spitale F.M., Torrisi F., Zappala A., Gulino R., Pasquinucci L., Chiechio S., Parenti C., Parenti R. (2020). Intercellular communication and ion channels in neuropathic pain chronicization. Inflamm. Res..

[16] Vicario N., Zappala A., Calabrese G., Gulino R., Parenti C., Gulisano M., Parenti R. (2017). Connexins in the Central Nervous System: Physiological Traits and Neuroprotective Targets. Front. Physiol..

[17] Willecke K., Eiberger J., Degen J., Eckardt D., Romualdi A., Guldenagel M., Deutsch U., Sohl G. (2002). Structural and functional diversity of connexin genes in the mouse and human genome. Biol. Chem..

[18] Bruzzone R., White T.W., Goodenough D.A. (1996). The cellular Internet: On-line with connexins. Bioessays.

[19] Retamal M.A., Froger N., Palacios-Prado N., Ezan P., Saez P.J., Saez J.C., Giaume C. (2007). Cx43 hemichannels and gap junction channels in astrocytes are regulated oppositely by proinflammatory cytokines released from activated microglia. J. Neurosci..

[20] Nijjar S., Maddison D., Meigh L., de Wolf E., Rodgers T., Cann M.J., Dale N. (2021). Opposing modulation of Cx26 gap junctions and hemichannels by CO_2_. J. Physiol..

[21] Khan A.K., Jagielnicki M., McIntire W.E., Purdy M.D., Dharmarajan V., Griffin P.R., Yeager M. (2020). A Steric ″Ball-and-Chain″ Mechanism for pH-Mediated Regulation of Gap Junction Channels. Cell Rep..

[22] De Vuyst E., Decrock E., De Bock M., Yamasaki H., Naus C.C., Evans W.H., Leybaert L. (2007). Connexin hemichannels and gap junction channels are differentially influenced by lipopolysaccharide and basic fibroblast growth factor. Mol. Biol. Cell.

[23] Zhao Y., Xin Y., He Z., Hu W. (2018). Function of Connexins in the Interaction between Glial and Vascular Cells in the Central Nervous System and Related Neurological Diseases. Neural Plast..

[24] Decrock E., De Bock M., Wang N., Bultynck G., Giaume C., Naus C.C., Green C.R., Leybaert L. (2015). Connexin and pannexin signaling pathways, an architectural blueprint for CNS physiology and pathology?. Cell. Mol. Life Sci..

[25] De Bock M., Leybaert L., Giaume C. (2017). Connexin Channels at the Glio-Vascular Interface: Gatekeepers of the Brain. Neurochem. Res..

[26] Zlokovic B.V. (2008). The blood-brain barrier in health and chronic neurodegenerative disorders. Neuron.

[27] Varatharaj A., Galea I. (2017). The blood-brain barrier in systemic inflammation. Brain Behav. Immun..

[28] Neuwelt E.A., Bauer B., Fahlke C., Fricker G., Iadecola C., Janigro D., Leybaert L., Molnar Z., O’Donnell M.E., Povlishock J.T. (2011). Engaging neuroscience to advance translational research in brain barrier biology. Nat. Rev. Neurosci..

[29] Simard M., Arcuino G., Takano T., Liu Q.S., Nedergaard M. (2003). Signaling at the gliovascular interface. J. Neurosci..

[30] Pannasch U., Vargova L., Reingruber J., Ezan P., Holcman D., Giaume C., Sykova E., Rouach N. (2011). Astroglial networks scale synaptic activity and plasticity. Proc. Natl. Acad. Sci. USA.

[31] Nakase T., Maeda T., Yoshida Y., Nagata K. (2009). Ischemia alters the expression of connexins in the aged human brain. J. Biomed. Biotechnol..

[32] Attwell D., Buchan A.M., Charpak S., Lauritzen M., Macvicar B.A., Newman E.A. (2010). Glial and neuronal control of brain blood flow. Nature.

[33] Stackhouse T.L., Mishra A. (2021). Neurovascular Coupling in Development and Disease: Focus on Astrocytes. Front. Cell Dev. Biol..

[34] Powers W.J., Hirsch I.B., Cryer P.E. (1996). Effect of stepped hypoglycemia on regional cerebral blood flow response to physiological brain activation. Am. J. Physiol..

[35] Lindauer U., Leithner C., Kaasch H., Rohrer B., Foddis M., Fuchtemeier M., Offenhauser N., Steinbrink J., Royl G., Kohl-Bareis M. (2010). Neurovascular coupling in rat brain operates independent of hemoglobin deoxygenation. J. Cereb. Blood Flow Metab..

[36] Buxton R.B. (2021). The thermodynamics of thinking: Connections between neural activity, energy metabolism and blood flow. Philos. Trans. R. Soc. B Biol. Sci..

[37] Rouach N., Koulakoff A., Giaume C. (2004). Neurons set the tone of gap junctional communication in astrocytic networks. Neurochem. Int..

[38] Giaume C., Koulakoff A., Roux L., Holcman D., Rouach N. (2010). Astroglial networks: A step further in neuroglial and gliovascular interactions. Nat. Rev. Neurosci..

[39] Charveriat M., Naus C.C., Leybaert L., Saez J.C., Giaume C. (2017). Connexin-Dependent Neuroglial Networking as a New Therapeutic Target. Front. Cell. Neurosci..

[40] Noctor S.C., Flint A.C., Weissman T.A., Wong W.S., Clinton B.K., Kriegstein A.R. (2002). Dividing precursor cells of the embryonic cortical ventricular zone have morphological and molecular characteristics of radial glia. J. Neurosci..

[41] Kilb W., Kirischuk S., Luhmann H.J. (2011). Electrical activity patterns and the functional maturation of the neocortex. Eur. J. Neurosci..

[42] Schock S.C., Leblanc D., Hakim A.M., Thompson C.S. (2008). ATP release by way of connexin 36 hemichannels mediates ischemic tolerance in vitro. Biochem. Biophys. Res. Commun..

[43] Moore A.R., Zhou W.L., Sirois C.L., Belinsky G.S., Zecevic N., Antic S.D. (2014). Connexin hemichannels contribute to spontaneous electrical activity in the human fetal cortex. Proc. Natl. Acad. Sci. USA.

[44] Parenti R., Gulisano M., Zappala A., Cicirata F. (2000). Expression of connexin36 mRNA in adult rodent brain. Neuroreport.

[45] Gulisano M., Parenti R., Spinella F., Cicirata F. (2000). Cx36 is dynamically expressed during early development of mouse brain and nervous system. Neuroreport.

[46] Condorelli D.F., Parenti R., Spinella F., Trovato Salinaro A., Belluardo N., Cardile V., Cicirata F. (1998). Cloning of a new gap junction gene (Cx36) highly expressed in mammalian brain neurons. Eur. J. Neurosci..

[47] Parpura V., Heneka M.T., Montana V., Oliet S.H., Schousboe A., Haydon P.G., Stout R.F., Spray D.C., Reichenbach A., Pannicke T. (2012). Glial cells in (patho)physiology. J. Neurochem..

[48] Barker A.J., Ullian E.M. (2008). New roles for astrocytes in developing synaptic circuits. Commun. Integr. Biol..

[49] Bramanti V., Grasso S., Tibullo D., Giallongo C., Pappa R., Brundo M.V., Tomassoni D., Viola M., Amenta F., Avola R. (2016). Neuroactive molecules and growth factors modulate cytoskeletal protein expression during astroglial cell proliferation and differentiation in culture. J. Neurosci. Res..

[50] Iadecola C., Nedergaard M. (2007). Glial regulation of the cerebral microvasculature. Nat. Neurosci..

[51] Haydon P.G., Carmignoto G. (2006). Astrocyte control of synaptic transmission and neurovascular coupling. Physiol. Rev..

[52] Araque A., Parpura V., Sanzgiri R.P., Haydon P.G. (1999). Tripartite synapses: Glia, the unacknowledged partner. Trends Neurosci..

[53] Orellana J.A., Stehberg J. (2014). Hemichannels: New roles in astroglial function. Front. Physiol..

[54] Eroglu C., Barres B.A. (2010). Regulation of synaptic connectivity by glia. Nature.

[55] Cotman C.W., Nieto-Sampedro M. (1984). Cell biology of synaptic plasticity. Science.

[56] Mazaud D., Capano A., Rouach N. (2021). The many ways astroglial connexins regulate neurotransmission and behavior. Glia.

[57] Cibelli A., Stout R., Timmermann A., de Menezes L., Guo P., Maass K., Seifert G., Steinhauser C., Spray D.C., Scemes E. (2021). Cx43 carboxyl terminal domain determines AQP4 and Cx30 endfoot organization and blood brain barrier permeability. Sci. Rep..

[58] Drewes L.R. (2012). Making connexons in the neurovascular unit. J. Cereb. Blood Flow Metab..

[59] Tanigami H., Okamoto T., Yasue Y., Shimaoka M. (2012). Astroglial integrins in the development and regulation of neurovascular units. Pain Res. Treat..

[60] Munoz M.F., Puebla M., Figueroa X.F. (2015). Control of the neurovascular coupling by nitric oxide-dependent regulation of astrocytic Ca(2+) signaling. Front. Cell. Neurosci..

[61] Theis M., Jauch R., Zhuo L., Speidel D., Wallraff A., Doring B., Frisch C., Sohl G., Teubner B., Euwens C. (2003). Accelerated hippocampal spreading depression and enhanced locomotory activity in mice with astrocyte-directed inactivation of connexin43. J. Neurosci..

[62] Lutz S.E., Zhao Y., Gulinello M., Lee S.C., Raine C.S., Brosnan C.F. (2009). Deletion of astrocyte connexins 43 and 30 leads to a dysmyelinating phenotype and hippocampal CA1 vacuolation. J. Neurosci..

[63] Ezan P., Andre P., Cisternino S., Saubamea B., Boulay A.C., Doutremer S., Thomas M.A., Quenech’du N., Giaume C., Cohen-Salmon M. (2012). Deletion of astroglial connexins weakens the blood-brain barrier. J. Cereb. Blood Flow Metab..

[64] Dere E., De Souza-Silva M.A., Frisch C., Teubner B., Sohl G., Willecke K., Huston J.P. (2003). Connexin30-deficient mice show increased emotionality and decreased rearing activity in the open-field along with neurochemical changes. Eur. J. Neurosci..

[65] Pannasch U., Derangeon M., Chever O., Rouach N. (2012). Astroglial gap junctions shape neuronal network activity. Commun. Integr. Biol..

[66] Hillen A.E.J., Burbach J.P.H., Hol E.M. (2018). Cell adhesion and matricellular support by astrocytes of the tripartite synapse. Prog. Neurobiol..

[67] Chever O., Pannasch U., Ezan P., Rouach N. (2014). Astroglial connexin 43 sustains glutamatergic synaptic efficacy. Philos. Trans. R Soc. B Biol. Sci..

[68] Ebong E.E., Depaola N. (2013). Specificity in the participation of connexin proteins in flow-induced endothelial gap junction communication. Pflugers Arch..

[69] Alvarez-Maubecin V., Garcia-Hernandez F., Williams J.T., Van Bockstaele E.J. (2000). Functional coupling between neurons and glia. J. Neurosci..

[70] Stout C.E., Costantin J.L., Naus C.C., Charles A.C. (2002). Intercellular calcium signaling in astrocytes via ATP release through connexin hemichannels. J. Biol. Chem..

[71] Striedinger K., Meda P., Scemes E. (2007). Exocytosis of ATP from astrocyte progenitors modulates spontaneous Ca2+ oscillations and cell migration. Glia.

[72] Boulay A.C., Saubamea B., Cisternino S., Mignon V., Mazeraud A., Jourdren L., Blugeon C., Cohen-Salmon M. (2015). The Sarcoglycan complex is expressed in the cerebrovascular system and is specifically regulated by astroglial Cx30 channels. Front. Cell. Neurosci..

[73] Giaume C., Leybaert L., Naus C.C., Saez J.C. (2013). Connexin and pannexin hemichannels in brain glial cells: Properties, pharmacology, and roles. Front. Pharmacol..

[74] Faustmann P.M., Haase C.G., Romberg S., Hinkerohe D., Szlachta D., Smikalla D., Krause D., Dermietzel R. (2003). Microglia activation influences dye coupling and Cx43 expression of the astrocytic network. Glia.

[75] Koulakoff A., Ezan P., Giaume C. (2008). Neurons control the expression of connexin 30 and connexin 43 in mouse cortical astrocytes. Glia.

[76] Wake H., Moorhouse A.J., Miyamoto A., Nabekura J. (2013). Microglia: Actively surveying and shaping neuronal circuit structure and function. Trends Neurosci..

[77] Wu Y., Dissing-Olesen L., MacVicar B.A., Stevens B. (2015). Microglia: Dynamic Mediators of Synapse Development and Plasticity. Trends Immunol..

[78] Csaszar E., Lenart N., Cserep C., Kornyei Z., Fekete R., Posfai B., Balazsfi D., Hangya B., Schwarcz A.D., Szabadits E. (2022). Microglia modulate blood flow, neurovascular coupling, and hypoperfusion via purinergic actions. J. Exp. Med..

[79] Gajardo-Gomez R., Labra V.C., Orellana J.A. (2016). Connexins and Pannexins: New Insights into Microglial Functions and Dysfunctions. Front. Mol. Neurosci..

[80] Schafer D.P., Lehrman E.K., Stevens B. (2013). The ″quad-partite″ synapse: Microglia-synapse interactions in the developing and mature CNS. Glia.

[81] Iadecola C. (2017). The Neurovascular Unit Coming of Age: A Journey through Neurovascular Coupling in Health and Disease. Neuron.

[82] Kisler K., Nelson A.R., Montagne A., Zlokovic B.V. (2017). Cerebral blood flow regulation and neurovascular dysfunction in Alzheimer disease. Nat.Rev. Neurosci..

[83] Parenti R., Campisi A., Vanella A., Cicirata F. (2002). Immunocytochemical and RT-PCR analysis of connexin36 in cultures of mammalian glial cells. Arch. Ital. Biol..

[84] Moon Y., Choi S.Y., Kim K., Kim H., Sun W. (2010). Expression of connexin29 and 32 in the penumbra region after traumatic brain injury of mice. Neuroreport.

[85] Eugenin E.A., Eckardt D., Theis M., Willecke K., Bennett M.V., Saez J.C. (2001). Microglia at brain stab wounds express connexin 43 and in vitro form functional gap junctions after treatment with interferon-gamma and tumor necrosis factor-alpha. Proc. Natl. Acad. Sci. USA.

[86] Saez P.J., Shoji K.F., Retamal M.A., Harcha P.A., Ramirez G., Jiang J.X., von Bernhardi R., Saez J.C. (2013). ATP is required and advances cytokine-induced gap junction formation in microglia in vitro. Mediat. Inflamm..

[87] Takeuchi H., Jin S., Wang J., Zhang G., Kawanokuchi J., Kuno R., Sonobe Y., Mizuno T., Suzumura A. (2006). Tumor necrosis factor-alpha induces neurotoxicity via glutamate release from hemichannels of activated microglia in an autocrine manner. J. Biol. Chem..

[88] Cepeda C., Chang J.W., Owens G.C., Huynh M.N., Chen J.Y., Tran C., Vinters H.V., Levine M.S., Mathern G.W. (2015). In Rasmussen encephalitis, hemichannels associated with microglial activation are linked to cortical pyramidal neuron coupling: A possible mechanism for cellular hyperexcitability. CNS Neurosci. Ther..

[89] Liu Y.D., Tang G., Qian F., Liu L., Huang J.R., Tang F.R. (2021). Astroglial Connexins in Neurological and Neuropsychological Disorders and Radiation Exposure. Curr. Med. Chem..

[90] Ma Y., Cao W., Wang L., Jiang J., Nie H., Wang B., Wei X., Ying W. (2014). Basal CD38/cyclic ADP-ribose-dependent signaling mediates ATP release and survival of microglia by modulating connexin 43 hemichannels. Glia.

[91] Orellana J.A., Figueroa X.F., Sanchez H.A., Contreras-Duarte S., Velarde V., Saez J.C. (2011). Hemichannels in the neurovascular unit and white matter under normal and inflamed conditions. CNS Neurol. Disord. Drug Targets.

[92] Parenti R., Cicirata F., Zappala A., Catania A., La Delia F., Cicirata V., Tress O., Willecke K. (2010). Dynamic expression of Cx47 in mouse brain development and in the cuprizone model of myelin plasticity. Glia.

[93] Dermietzel R., Farooq M., Kessler J.A., Althaus H., Hertzberg E.L., Spray D.C. (1997). Oligodendrocytes express gap junction proteins connexin32 and connexin45. Glia.

[94] Eiberger J., Kibschull M., Strenzke N., Schober A., Bussow H., Wessig C., Djahed S., Reucher H., Koch D.A., Lautermann J. (2006). Expression pattern and functional characterization of connexin29 in transgenic mice. Glia.

[95] Kunzelmann P., Blumcke I., Traub O., Dermietzel R., Willecke K. (1997). Coexpression of connexin45 and -32 in oligodendrocytes of rat brain. J. Neurocytol..

[96] Menichella D.M., Goodenough D.A., Sirkowski E., Scherer S.S., Paul D.L. (2003). Connexins are critical for normal myelination in the CNS. J. Neurosci..

[97] Nagy J.I., Dudek F.E., Rash J.E. (2004). Update on connexins and gap junctions in neurons and glia in the mammalian nervous system. Brain Res. Rev..

[98] Nagy J.I., Ionescu A.V., Lynn B.D., Rash J.E. (2003). Coupling of astrocyte connexins Cx26, Cx30, Cx43 to oligodendrocyte Cx29, Cx32, Cx47: Implications from normal and connexin32 knockout mice. Glia.

[99] Maglione M., Tress O., Haas B., Karram K., Trotter J., Willecke K., Kettenmann H. (2010). Oligodendrocytes in mouse corpus callosum are coupled via gap junction channels formed by connexin47 and connexin32. Glia.

[100] Orthmann-Murphy J.L., Freidin M., Fischer E., Scherer S.S., Abrams C.K. (2007). Two distinct heterotypic channels mediate gap junction coupling between astrocyte and oligodendrocyte connexins. J. Neurosci..

[101] Kamasawa N., Sik A., Morita M., Yasumura T., Davidson K.G., Nagy J.I., Rash J.E. (2005). Connexin-47 and connexin-32 in gap junctions of oligodendrocyte somata, myelin sheaths, paranodal loops and Schmidt-Lanterman incisures: Implications for ionic homeostasis and potassium siphoning. Neuroscience.

[102] Menichella D.M., Majdan M., Awatramani R., Goodenough D.A., Sirkowski E., Scherer S.S., Paul D.L. (2006). Genetic and physiological evidence that oligodendrocyte gap junctions contribute to spatial buffering of potassium released during neuronal activity. J. Neurosci..

[103] Niu J., Li T., Yi C., Huang N., Koulakoff A., Weng C., Li C., Zhao C.J., Giaume C., Xiao L. (2016). Connexin-based channels contribute to metabolic pathways in the oligodendroglial lineage. J. Cell Sci..

[104] Arai K., Lo E.H. (2009). An oligovascular niche: Cerebral endothelial cells promote the survival and proliferation of oligodendrocyte precursor cells. J. Neurosci..

[105] Hamanaka G., Ohtomo R., Takase H., Lok J., Arai K. (2018). White-matter repair: Interaction between oligodendrocytes and the neurovascular unit. Brain Circ..

[106] Seo J.H., Maki T., Maeda M., Miyamoto N., Liang A.C., Hayakawa K., Pham L.D., Suwa F., Taguchi A., Matsuyama T. (2014). Oligodendrocyte precursor cells support blood-brain barrier integrity via TGF-beta signaling. PLoS ONE.

[107] Wang Q., Wang Z., Tian Y., Zhang H., Fang Y., Yu Z., Wang W., Xie M., Ding F. (2018). Inhibition of Astrocyte Connexin 43 Channels Facilitates the Differentiation of Oligodendrocyte Precursor Cells Under Hypoxic Conditions In Vitro. J. Mol. Neurosci..

[108] Keep R.F., Andjelkovic A.V., Xiang J., Stamatovic S.M., Antonetti D.A., Hua Y., Xi G. (2018). Brain endothelial cell junctions after cerebral hemorrhage: Changes, mechanisms and therapeutic targets. J. Cereb. Blood Flow Metab..

[109] Hautefort A., Pfenniger A., Kwak B.R. (2019). Endothelial connexins in vascular function. Vasc. Biol..

[110] Hogan-Cann A.D., Lu P., Anderson C.M. (2019). Endothelial NMDA receptors mediate activity-dependent brain hemodynamic responses in mice. Proc. Natl. Acad. Sci. USA.

[111] Stobart J.L., Lu L., Anderson H.D., Mori H., Anderson C.M. (2013). Astrocyte-induced cortical vasodilation is mediated by D-serine and endothelial nitric oxide synthase. Proc. Natl. Acad. Sci. USA.

[112] Zuccolo E., Lim D., Kheder D.A., Perna A., Catarsi P., Botta L., Rosti V., Riboni L., Sancini G., Tanzi F. (2017). Acetylcholine induces intracellular Ca(2+) oscillations and nitric oxide release in mouse brain endothelial cells. Cell Calcium.

[113] Okamoto T., Akiyama M., Takeda M., Gabazza E.C., Hayashi T., Suzuki K. (2009). Connexin32 is expressed in vascular endothelial cells and participates in gap-junction intercellular communication. Biochem. Biophys. Res. Commun..

[114] Okamoto T., Akiyama M., Takeda M., Akita N., Yoshida K., Hayashi T., Suzuki K. (2011). Connexin32 protects against vascular inflammation by modulating inflammatory cytokine expression by endothelial cells. Exp. Cell Res..

[115] Sokoya E.M., Burns A.R., Setiawan C.T., Coleman H.A., Parkington H.C., Tare M. (2006). Evidence for the involvement of myoendothelial gap junctions in EDHF-mediated relaxation in the rat middle cerebral artery. Am. J. Physiol. Heart Circ. Physiol..

[116] Seul K.H., Kang K.Y., Lee K.S., Kim S.H., Beyer E.C. (2004). Adenoviral delivery of human connexin37 induces endothelial cell death through apoptosis. Biochem. Biophys. Res. Commun..

[117] Pogoda K., Fuller M., Pohl U., Kameritsch P. (2014). NO, via its target Cx37, modulates calcium signal propagation selectively at myoendothelial gap junctions. Cell Commun. Signal..

[118] Arthur F.E., Shivers R.R., Bowman P.D. (1987). Astrocyte-mediated induction of tight junctions in brain capillary endothelium: An efficient in vitro model. Brain Res..

[119] Rubin L.L., Hall D.E., Porter S., Barbu K., Cannon C., Horner H.C., Janatpour M., Liaw C.W., Manning K., Morales J. (1991). A cell culture model of the blood-brain barrier. J. Cell Biol..

[120] Cibelli A., Veronica Lopez-Quintero S., McCutcheon S., Scemes E., Spray D.C., Stout R.F., Suadicani S.O., Thi M.M., Urban-Maldonado M. (2021). Generation and Characterization of Immortalized Mouse Cortical Astrocytes From Wildtype and Connexin43 Knockout Mice. Front. Cell. Neurosci..

[121] Wuestefeld R., Chen J., Meller K., Brand-Saberi B., Theiss C. (2012). Impact of vegf on astrocytes: Analysis of gap junctional intercellular communication, proliferation, and motility. Glia.

[122] Yu W., Jin H., Sun W., Nan D., Deng J., Jia J., Yu Z., Huang Y. (2021). Connexin43 promotes angiogenesis through activating the HIF-1alpha/VEGF signaling pathway under chronic cerebral hypoperfusion. J. Cereb. Blood Flow Metab..

[123] Chen J., Gu Z., Wu M., Yang Y., Zhang J., Ou J., Zuo Z., Wang J., Chen Y. (2016). C-reactive protein can upregulate VEGF expression to promote ADSC-induced angiogenesis by activating HIF-1alpha via CD64/PI3k/Akt and MAPK/ERK signaling pathways. Stem Cell Res. Ther..

[124] Asahara T., Takahashi T., Masuda H., Kalka C., Chen D., Iwaguro H., Inai Y., Silver M., Isner J.M. (1999). VEGF contributes to postnatal neovascularization by mobilizing bone marrow-derived endothelial progenitor cells. EMBO J..

[125] Gartner C., Ziegelhoffer B., Kostelka M., Stepan H., Mohr F.W., Dhein S. (2012). Knock-down of endothelial connexins impairs angiogenesis. Pharmacol. Res..

[126] Li A., Cho J.H., Reid B., Tseng C.C., He L., Tan P., Yeh C.Y., Wu P., Li Y., Widelitz R.B. (2018). Calcium oscillations coordinate feather mesenchymal cell movement by SHH dependent modulation of gap junction networks. Nat. Commun..

[127] Winkler E.A., Bell R.D., Zlokovic B.V. (2011). Central nervous system pericytes in health and disease. Nat. Neurosci..

[128] Daneman R., Zhou L., Kebede A.A., Barres B.A. (2010). Pericytes are required for blood-brain barrier integrity during embryogenesis. Nature.

[129] Dore-Duffy P. (2008). Pericytes: Pluripotent cells of the blood brain barrier. Curr. Pharm. Des..

[130] Uemura M.T., Maki T., Ihara M., Lee V.M.Y., Trojanowski J.Q. (2020). Brain Microvascular Pericytes in Vascular Cognitive Impairment and Dementia. Front. Aging Neurosci..

[131] Attwell D., Mishra A., Hall C.N., O’Farrell F.M., Dalkara T. (2016). What is a pericyte?. J. Cereb. Blood Flow Metab..

[132] Payne L.B., Tewari B.P., Dunkenberger L., Bond S., Savelli A., Darden J., Zhao H., Willi C., Kanodia R., Gude R. (2022). Pericyte Progenitor Coupling to the Emerging Endothelium During Vasculogenesis via Connexin 43. Arter. Thromb. Vasc. Biol..

[133] Perrot C.Y., Herrera J.L., Fournier-Goss A.E., Komatsu M. (2020). Prostaglandin E2 breaks down pericyte-endothelial cell interaction via EP1 and EP4-dependent downregulation of pericyte N-cadherin, connexin-43, and R-Ras. Sci. Rep..

[134] Mazare N., Gilbert A., Boulay A.C., Rouach N., Cohen-Salmon M. (2018). Connexin 30 is expressed in a subtype of mouse brain pericytes. Brain Struct. Funct..

[135] Silva M.E., Lange S., Hinrichsen B., Philp A.R., Reyes C.R., Halabi D., Mansilla J.B., Rotheneichner P., Guzman de la Fuente A., Couillard-Despres S. (2019). Pericytes Favor Oligodendrocyte Fate Choice in Adult Neural Stem Cells. Front. Cell Neurosci..

[136] De La Fuente A.G., Lange S., Silva M.E., Gonzalez G.A., Tempfer H., van Wijngaarden P., Zhao C., Di Canio L., Trost A., Bieler L. (2017). Pericytes Stimulate Oligodendrocyte Progenitor Cell Differentiation during CNS Remyelination. Cell Rep..

[137] Muoio V., Persson P.B., Sendeski M.M. (2014). The neurovascular unit-concept review. Acta Physiol..

[138] Quelhas P., Baltazar G., Cairrao E. (2019). The Neurovascular Unit: Focus on the Regulation of Arterial Smooth Muscle Cells. Curr. Neurovasc. Res..

[139] Pan Q., He C., Liu H., Liao X., Dai B., Chen Y., Yang Y., Zhao B., Bihl J., Ma X. (2016). Microvascular endothelial cells-derived microvesicles imply in ischemic stroke by modulating astrocyte and blood brain barrier function and cerebral blood flow. Mol. Brain.

[140] Filosa J.A., Morrison H.W., Iddings J.A., Du W., Kim K.J. (2016). Beyond neurovascular coupling, role of astrocytes in the regulation of vascular tone. Neuroscience.

[141] Mariana M., Roque C., Baltazar G., Cairrao E. (2021). In Vitro Model for Ischemic Stroke: Functional Analysis of Vascular Smooth Muscle Cells. Cell. Mol. Neurobiol..

[142] Filiberto A.C., Spinosa M.D., Elder C.T., Su G., Leroy V., Ladd Z., Lu G., Mehaffey J.H., Salmon M.D., Hawkins R.B. (2022). Endothelial pannexin-1 channels modulate macrophage and smooth muscle cell activation in abdominal aortic aneurysm formation. Nat. Commun..

[143] Qin X., He W., Yang R., Liu L., Zhang Y., Li L., Si J., Li X., Ma K. (2022). Inhibition of Connexin 43 reverses ox-LDL-mediated inhibition of autophagy in VSMC by inhibiting the PI3K/Akt/mTOR signaling pathway. PeerJ.

[144] Liu W., Li Y., Zhao D. (2021). Modulation of Vascular Smooth Muscle Cell Multiplication, Apoptosis, and Inflammatory Damage by miR-21 in Coronary Heart Disease. Comput. Math. Methods Med..

[145] Faissner A., Pyka M., Geissler M., Sobik T., Frischknecht R., Gundelfinger E.D., Seidenbecher C. (2010). Contributions of astrocytes to synapse formation and maturation-Potential functions of the perisynaptic extracellular matrix. Brain Res. Rev..

[146] Michalski D., Spielvogel E., Puchta J., Reimann W., Barthel H., Nitzsche B., Mages B., Jager C., Martens H., Horn A.K.E. (2020). Increased Immunosignals of Collagen IV and Fibronectin Indicate Ischemic Consequences for the Neurovascular Matrix Adhesion Zone in Various Animal Models and Human Stroke Tissue. Front. Physiol..

[147] Lu P., Takai K., Weaver V.M., Werb Z. (2011). Extracellular matrix degradation and remodeling in development and disease. Cold Spring Harb. Perspect. Biol..

[148] Del Zoppo G.J., Milner R., Mabuchi T., Hung S., Wang X., Koziol J.A. (2006). Vascular matrix adhesion and the blood-brain barrier. Biochem. Soc. Trans..

[149] Kim S.H., Turnbull J., Guimond S. (2011). Extracellular matrix and cell signalling: The dynamic cooperation of integrin, proteoglycan and growth factor receptor. J. Endocrinol..

[150] Thomsen M.S., Routhe L.J., Moos T. (2017). The vascular basement membrane in the healthy and pathological brain. J. Cereb. Blood Flow Metab..

[151] Gatseva A., Sin Y.Y., Brezzo G., Van Agtmael T. (2019). Basement membrane collagens and disease mechanisms. Essays Biochem..

[152] Reed M.J., Damodarasamy M., Banks W.A. (2019). The extracellular matrix of the blood-brain barrier: Structural and functional roles in health, aging, and Alzheimer’s disease. Tissue Barriers.

[153] Xu L., Nirwane A., Yao Y. (2019). Basement membrane and blood-brain barrier. Stroke Vasc. Neurol..

[154] Andrade-Rozental A.F., Rozental R., Hopperstad M.G., Wu J.K., Vrionis F.D., Spray D.C. (2000). Gap junctions: The ″kiss of death″ and the ″kiss of life″. Brain Res. Rev..

[155] Spray D.C., Hanstein R., Lopez-Quintero S.V., Stout R.F., Suadicani S.O., Thi M.M. (2013). Gap junctions and Bystander Effects: Good Samaritans and executioners. Wiley Interdiscip. Rev. Membr. Transp. Signal..

[156] Decrock E., Vinken M., De Vuyst E., Krysko D.V., D’Herde K., Vanhaecke T., Vandenabeele P., Rogiers V., Leybaert L. (2009). Connexin-related signaling in cell death: To live or let die?. Cell Death Differ..

[157] Dermietzel R., Gao Y., Scemes E., Vieira D., Urban M., Kremer M., Bennett M.V., Spray D.C. (2000). Connexin43 null mice reveal that astrocytes express multiple connexins. Brain Res. Rev..

[158] Rouach N., Avignone E., Meme W., Koulakoff A., Venance L., Blomstrand F., Giaume C. (2002). Gap junctions and connexin expression in the normal and pathological central nervous system. Biol. Cell.

[159] Giaume C., Naus C.C., Saez J.C., Leybaert L. (2021). Glial Connexins and Pannexins in the Healthy and Diseased Brain. Physiol. Rev..

[160] Zhang Y., Khan S., Liu Y., Siddique R., Zhang R., Yong V.W., Xue M. (2021). Gap Junctions and Hemichannels Composed of Connexins and Pannexins Mediate the Secondary Brain Injury Following Intracerebral Hemorrhage. Biology.

[161] Xue M., Yong V.W. (2020). Neuroinflammation in intracerebral haemorrhage: Immunotherapies with potential for translation. Lancet Neurol..

[162] Yu H., Cao X., Li W., Liu P., Zhao Y., Song L., Chen J., Chen B., Yu W., Xu Y. (2020). Targeting connexin 43 provides anti-inflammatory effects after intracerebral hemorrhage injury by regulating YAP signaling. J. Neuroinflammation.

[163] Kim Y., Davidson J.O., Gunn K.C., Phillips A.R., Green C.R., Gunn A.J. (2016). Role of Hemichannels in CNS Inflammation and the Inflammasome Pathway. Adv. Protein Chem. Struct. Biol..

[164] Brand-Schieber E., Werner P., Iacobas D.A., Iacobas S., Beelitz M., Lowery S.L., Spray D.C., Scemes E. (2005). Connexin43, the major gap junction protein of astrocytes, is down-regulated in inflamed white matter in an animal model of multiple sclerosis. J. Neurosci. Res..

[165] Markoullis K., Sargiannidou I., Gardner C., Hadjisavvas A., Reynolds R., Kleopa K.A. (2012). Disruption of oligodendrocyte gap junctions in experimental autoimmune encephalomyelitis. Glia.

[166] Mei X., Ezan P., Giaume C., Koulakoff A. (2010). Astroglial connexin immunoreactivity is specifically altered at beta-amyloid plaques in beta-amyloid precursor protein/presenilin1 mice. Neuroscience.

[167] Spitale F.M., Vicario N., Rosa M.D., Tibullo D., Vecchio M., Gulino R., Parenti R. (2020). Increased expression of connexin 43 in a mouse model of spinal motoneuronal loss. Aging.

[168] Almad A.A., Doreswamy A., Gross S.K., Richard J.P., Huo Y., Haughey N., Maragakis N.J. (2016). Connexin 43 in astrocytes contributes to motor neuron toxicity in amyotrophic lateral sclerosis. Glia.

[169] Almad A.A., Taga A., Joseph J., Gross S.K., Welsh C., Patankar A., Richard J.P., Rust K., Pokharel A., Plott C. (2022). Cx43 hemichannels contribute to astrocyte-mediated toxicity in sporadic and familial ALS. Proc. Natl. Acad. Sci. USA.

[170] Harcha P.A., Garces P., Arredondo C., Fernandez G., Saez J.C., van Zundert B. (2021). Mast Cell and Astrocyte Hemichannels and Their Role in Alzheimer’s Disease, ALS, and Harmful Stress Conditions. Int. J. Mol. Sci..

[171] Chen Y., Wang L., Zhang L., Chen B., Yang L., Li X., Li Y., Yu H. (2018). Inhibition of Connexin 43 Hemichannels Alleviates Cerebral Ischemia/Reperfusion Injury via the TLR4 Signaling Pathway. Front. Cell. Neurosci..

[172] Chen B., Yang L., Chen J., Chen Y., Zhang L., Wang L., Li X., Li Y., Yu H. (2019). Inhibition of Connexin43 hemichannels with Gap19 protects cerebral ischemia/reperfusion injury via the JAK2/STAT3 pathway in mice. Brain Res. Bull..

[173] Zhou Z., Wei X., Xiang J., Gao J., Wang L., You J., Cai Y., Cai D. (2015). Protection of erythropoietin against ischemic neurovascular unit injuries through the effects of connexin43. Biochem. Biophys. Res. Commun..

[174] Freitas-Andrade M., Bechberger J., Wang J., Yeung K.K.C., Whitehead S.N., Hansen R.S., Naus C.C. (2020). Danegaptide Enhances Astrocyte Gap Junctional Coupling and Reduces Ischemic Reperfusion Brain Injury in Mice. Biomolecules.

[175] Bhowmick S., D’Mello V., Caruso D., Wallerstein A., Abdul-Muneer P.M. (2019). Impairment of pericyte-endothelium crosstalk leads to blood-brain barrier dysfunction following traumatic brain injury. Exp. Neurol..

[176] Eleftheriou C.G., Ivanova E., Sagdullaev B.T. (2020). Of neurons and pericytes: The neuro-vascular approach to diabetic retinopathy. Vis. Neurosci..

[177] Nakase T., Sohl G., Theis M., Willecke K., Naus C.C. (2004). Increased apoptosis and inflammation after focal brain ischemia in mice lacking connexin43 in astrocytes. Am. J. Pathol..

[178] Xie M., Yi C., Luo X., Xu S., Yu Z., Tang Y., Zhu W., Du Y., Jia L., Zhang Q. (2011). Glial gap junctional communication involvement in hippocampal damage after middle cerebral artery occlusion. Ann. Neurol..

[179] Freitas A.S., Xavier A.L., Furtado C.M., Hedin-Pereira C., Froes M.M., Menezes J.R. (2012). Dye coupling and connexin expression by cortical radial glia in the early postnatal subventricular zone. Dev. Neurobiol..

[180] Liu X., Bolteus A.J., Balkin D.M., Henschel O., Bordey A. (2006). GFAP-expressing cells in the postnatal subventricular zone display a unique glial phenotype intermediate between radial glia and astrocytes. Glia.

[181] Li T., Niu J., Yu G., Ezan P., Yi C., Wang X., Koulakoff A., Gao X., Chen X., Saez J.C. (2020). Connexin 43 deletion in astrocytes promotes CNS remyelination by modulating local inflammation. Glia.

[182] Glen C.M., McDevitt T.C., Kemp M.L. (2018). Dynamic intercellular transport modulates the spatial patterning of differentiation during early neural commitment. Nat. Commun..

[183] Morioka N., Nakamura Y., Zhang F.F., Hisaoka-Nakashima K., Nakata Y. (2019). Role of Connexins in Chronic Pain and Their Potential as Therapeutic Targets for Next-Generation Analgesics. Biol. Pharm. Bull..

[184] Vicario N., Pasquinucci L., Spitale F.M., Chiechio S., Turnaturi R., Caraci F., Tibullo D., Avola R., Gulino R., Parenti R. (2019). Simultaneous Activation of Mu and Delta Opioid Receptors Reduces Allodynia and Astrocytic Connexin 43 in an Animal Model of Neuropathic Pain. Mol. Neurobiol..

[185] Vicario N., Denaro S., Turnaturi R., Longhitano L., Spitale F.M., Spoto S., Marrazzo A., Zappala A., Tibullo D., Li Volti G. (2022). Mu and Delta Opioid Receptor Targeting Reduces Connexin 43-Based Heterocellular Coupling during Neuropathic Pain. Int. J. Mol. Sci..

[186] Torrisi F., Alberghina C., D’Aprile S., Pavone A.M., Longhitano L., Giallongo S., Tibullo D., Di Rosa M., Zappala A., Cammarata F.P. (2022). The Hallmarks of Glioblastoma: Heterogeneity, Intercellular Crosstalk and Molecular Signature of Invasiveness and Progression. Biomedicines.

[187] Torrisi F., Alberghina C., Lo Furno D., Zappala A., Valable S., Li Volti G., Tibullo D., Vicario N., Parenti R. (2021). Connexin 43 and Sonic Hedgehog Pathway Interplay in Glioblastoma Cell Proliferation and Migration. Biology.

[188] Sin W.C., Aftab Q., Bechberger J.F., Leung J.H., Chen H., Naus C.C. (2016). Astrocytes promote glioma invasion via the gap junction protein connexin43. Oncogene.

[189] Murphy S.F., Varghese R.T., Lamouille S., Guo S., Pridham K.J., Kanabur P., Osimani A.M., Sharma S., Jourdan J., Rodgers C.M. (2016). Connexin 43 Inhibition Sensitizes Chemoresistant Glioblastoma Cells to Temozolomide. Cancer Res..

[190] Aasen T., Mesnil M., Naus C.C., Lampe P.D., Laird D.W. (2016). Gap junctions and cancer: Communicating for 50 years. Nat. Rev. Cancer.

